# Analysis of the HTLV-1 Gag assembly pathway by biophysical fluorescence

**DOI:** 10.1186/1742-4690-8-S1-A206

**Published:** 2011-06-06

**Authors:** Keir H Fogarty, Yan Chen, Iwen F Grigsby, Patrick J Macdonald, Elizabeth M Smith, Jolene L Johnson, Jonathan M Rawson, Joachim D Mueller, Louis M Mansky

**Affiliations:** 1 Institute for Molecular Virology, University of Minnesota, Minneapolis, MN, 55455, USA; 2 School of Physics and Astronomy, University of Minnesota, Minneapolis, MN, 55455 USA

## 

Much of the mechanistic details for how HTLV-1 Gag orchestrates virus particle assembly and release are poorly understood. Here, we monitored the behavior of both membrane-bound and cytoplasmic HTLV-1 Gag in real-time in living cells incubated on a fluorescence microscope. We used both fluorescence fluctuation spectroscopy (FFS, conventional and z-scan) and fluorescence imaging (epi-illumination, total internal reflection fluorescence (TIRF)) to investigate the relationship between cytoplasmic and membrane bound Gag, using a Gag-YFP model system. FFS determines the brightness, mobility, and concentration (conventional) and localization (z-scan) of fluorescent particles from the intensity bursts generated by individual particles passing through a small observation volume, which yields information about protein stoichiometry, interactions, transport, and distribution. By coupling the single-molecule FFS technique with imaging techniques capable of monitoring Gag localization (epi-illumination) and membrane-specific localization (TIRF), we achieved new insights into the earliest events in HTLV-1 Gag assembly, and differences to HIV-1 Gag. We found that HTLV-1 Gag membrane-targeting occurred at all cytoplasmic concentrations measured, while appreciable membrane-targeting for HIV-1 required Gag cytoplasmic concentration to exceed a threshold. In addition, z-scan FFS revealed that a substantial population of membrane-bound HTLV-1 Gag exists not as puncta, but as a diffuse, low-order, dynamic “sheet.” These observations, coupled with previous observations of cytoplasmic Gag interactions and mobility, point to differences in membrane targeting of HTLV-1 and HIV-1 Gag. In summary, the suite of biophysical fluorescence techniques, applied HTLV-1 Gag, provide unparalleled information concerning HTLV-1 Gag trafficking processes in vivo, elucidating assembly pathway differences between HTLV-1 and other retroviruses.

